# Rates and Timing of Follow-up Colonoscopy After a Positive Stool-Based Test in an Integrated Health System

**DOI:** 10.1007/s11606-026-10226-8

**Published:** 2026-02-26

**Authors:** Christina P. Wang, Ankit M. Shah, Matthew Y. Zhao, Ji Yoon Yoon, Michelle Zhao, Yuhong Yang, Tsion Tmariam, Lina H. Jandorf, Steven H. Itzkowitz

**Affiliations:** 1 Division of Gastroenterology, Icahn School of Medicine at Mount Sinai, New York City, USA; 2 Department of Medicine, Icahn School of Medicine at Mount Sinai, New York City, USA; 3 Department of Medicine, Mount Sinai Morningside and Mount Sinai West, New York City, USA; 4 Department of Population Health Science and Policy, Icahn School of Medicine at Mount Sinai, New York City, USA

**Keywords:** Early detection of cancer, Colorectal neoplasms, Colonoscopy, Occult blood

## Abstract

**Background:**

Completion of follow-up colonoscopy after a positive stool-based test varies across health settings. Colonoscopy performed > 6 months following a positive stool test is associated with adverse colorectal cancer outcomes.

**Methods:**

This retrospective study included 701 patients aged 45–75 in an urban, integrated health system with a positive stool test between February 1, 2022, and January 31, 2023. We examined rates of timely (i.e., within 180 days) follow-up colonoscopy and at varying time points (90 days, 365 days, and any time during our follow-up period). Multivariable Cox proportional hazard models examined factors associated with timely colonoscopy, and a Pareto analysis identified barriers to timely completion.

**Results:**

The median age of this cohort was 64 years (IQR 56–70); 52.1% were female, 48.2% were non-Hispanic White, and 52.4% had a Charlson Comorbidity Index score ≥ 3. The rate of timely follow-up colonoscopy was 59.6%, with rates of 44.5% and 68.5% at 90 days and 365 days, respectively. In Cox models, patient outreach increased timely colonoscopy by 52% (HR 1.52, 95% CI 1.21–1.91), while Direct Access patients were less likely to complete timely colonoscopy (HR 0.59, 95% CI 0.41–0.86); no associations were observed with sociodemographic factors. The most common barriers to timely colonoscopy were (1) lack of gastroenterology clinic visit, (2) patient refusal, and (3) colonoscopy no-show or cancellation.

**Conclusions:**

The rate of timely, follow-up colonoscopy in this older, sicker population is suboptimal. System-level factors impact timely completion. A Pareto analysis reveals multiple elements that contribute to delays in colonoscopy and can inform interventions.

**Supplementary Information:**

The online version contains supplementary material available at 10.1007/s11606-026-10226-8.

## INTRODUCTION

Timely, follow-up colonoscopy to evaluate positive (abnormal) stool tests has been recognized as a priority by national organizations when discussing colorectal cancer (CRC) screening benchmarks.^1,2^ The US Multi-Society Task Force on Colorectal Cancer recommends an 80% target for completion of colonoscopy after a positive stool-based test; however, there are no formal guidelines regarding the timing of colonoscopy.^2^ Prior studies demonstrate higher CRC incidence and mortality rates when the time to follow-up colonoscopy is prolonged (especially beyond 6 months).^3–6^ Real-world practices reveal disparities in colonoscopy completion worsened by COVID-19 disruptions.^7–10^ Multiple barriers have been identified at the (1) patient-level, such as lack of transportation and fear of colonoscopy; (2) provider-level, including limited time to address due to competing medical priorities and knowledge gaps regarding the importance of follow-up colonoscopy; and (3) system-level, such as fragmented coordination between primary care and gastroenterology and lack of appointment reminders.^11,12^

Non-invasive stool tests are increasingly favored among unscreened, sicker, and minoritized populations and represent an equitable strategy for addressing the screening gap in these communities.^13,14^ During the COVID-19 pandemic, and in contrast to observed decreases associated with breast, cervical, and prostate cancer screenings, CRC screening rates remained steady due to a rise in at-home stool testing.^15,16^ As such, we sought to evaluate the rate of timely colonoscopy completion after a positive stool-based test and its associated factors across an urban, integrated health system.

## METHODS

Our project was deemed a quality project by the Quality Improvement Committee in the Department of Medicine at Mount Sinai Hospital, and thus an institutional review board submission was not required.

### Study Design and Population

The Mount Sinai Health System (MSHS) is an integrated system that consists of eight affiliated hospitals in the greater New York area, with more than 400 ambulatory practice locations throughout the New York City metropolitan and surrounding areas. We queried the electronic medical record (EMR) to identify all individuals aged 45–75 years in the MSHS with a positive stool-based result, using a fecal occult blood test (FOBT), fecal immunochemical test (FIT), or multi-target stool DNA test, from February 1, 2022, through January 31, 2023. We included patients with a positive stool test from any MSHS-affiliated ambulatory practice (primary or specialty care). We excluded patients who received stool tests in the inpatient or emergency room setting as they were likely performed for diagnostic purposes (e.g., evaluation of anemia). The follow-up period was from the date of the positive stool result through November 29, 2024. The study period was selected to evaluate recovery from the COVID-19 pandemic with respect to endoscopy restrictions and to allow for at least 1-year follow-up for colonoscopy completion. Each chart was manually reviewed by one of six abstractors to collect additional clinical and colonoscopy data as described below. Reviewers met on a weekly basis to discuss and resolve discrepancies and to ensure consistency in data collection and categorization.

Sociodemographic and clinical data, such as age, sex, race/ethnicity, language, insurance status, body mass index (BMI), and number of ambulatory visits in the year preceding the positive stool result, were queried from the EMR. We manually extracted additional clinical data as follows: Charlson Comorbidity Index (CCI) score, history of CRC screening, and whether patients were seen in gastroenterology (GI) clinic or enrolled in a Direct Access colonoscopy screening program following the positive result. The Direct Access program is a patient navigation system offered at certain ambulatory practices to expedite colonoscopy appointments by allowing patients to bypass in-office GI evaluations (details described separately^17^). CCI scores were calculated based on ICD-9 and ICD-10 codes from the office visit associated with the positive stool test. A history of CRC screening was defined as any prior FIT, FOBT, multi-target stool DNA, flexible sigmoidoscopy, standard colonoscopy, or virtual colonoscopy that preceded the positive stool test.

We recorded the first colonoscopy date following the positive stool result. Timely colonoscopy completion was defined as a colonoscopy or virtual colonoscopy performed within 180 days of a positive stool result. Virtual colonoscopy (i.e., CT colonography) was used if patients were too sick for conventional colonoscopy or if the procedure could not be technically completed. Colonoscopies performed outside of MSHS were included, provided that documentation was available in the EMR in the form of endoscopy reports, scanned external reports, or provider notes indicating a colonoscopy was performed. In the event of multiple colonoscopies performed following the positive stool test, the earliest, adequate, and complete colonoscopy was used to assess timeliness. Patients without clear evidence of endoscopic evaluation were considered “not completed.”

For colonoscopies without evidence of completion within 180 days of the positive stool result, we recorded the primary reason for colonoscopy delay. The primary reason for delay in timely colonoscopy was determined based on chart review and group discussion using categories defined a priori. The primary reason represented the most direct factor preventing timely completion. For example, if a colonoscopy was not scheduled because a GI clinic visit was not completed, the delay was attributed to “GI clinic visit not scheduled, no-show, or cancelled.”

We also collected colonoscopy outreach data, such as the number of outreach attempts, proof of successful outreach, and the date of last outreach. Outreach attempts were defined as patient contact *after* colonoscopy scheduling but *before* the procedure, and included EMR messages, phone calls, texts, or emails addressing reminders, bowel preparation, or procedural questions. “Successful” patient outreach was defined as evidence confirming message receipt or direct patient contact (e.g., “seen” status on EMR messages, documentation of successful telephone conversations, and email confirmations). “Last” outreach was defined as the date of the final successful reminder before the scheduled colonoscopy.

### Statistical Analysis

We used descriptive statistics for demographic and clinical characteristics of the study cohort. We used univariate and multivariable Cox proportional hazards models to assess the association between patient- and system-level covariates and colonoscopy completion; we report our results as hazard ratios (HRs) with 95% confidence intervals (95% CI). Our primary outcome was the rate of colonoscopy completion within 180 days of a positive stool-based test (henceforth, “timely colonoscopy”), given the increased risk of adverse CRC outcomes associated with colonoscopy delay beyond 6 months.^4,5^ Our secondary outcomes were the rates of colonoscopy completion within 90 days, 365 days, and at any time during follow-up after the positive stool result. For the multivariable Cox model, covariates were selected a priori based on factors associated with colonoscopy completion (i.e., age, sex, race/ethnicity, insurance status, comorbidity burden) as identified in the literature, as well as variables pertinent to the clinical workflow (i.e., GI clinic attendance, Direct Access enrollment, successful patient outreach).^7–9,18–20^ We also performed sensitivity analyses to evaluate the aforementioned covariates with respect to colonoscopy completion defined at different time periods. These analyses were conducted on SAS 9.4 (SAS Institute Inc. Cary, NC). Lastly, we conducted a Pareto analysis in Microsoft Excel to assess barriers to timely colonoscopy completion.

## RESULTS

### Cohort Characteristics

A total of 701 individuals aged 45–75 years with a positive stool-based result between February 1, 2022, and January 31, 2023, were included. Sociodemographic and clinical characteristics are outlined in Table 1. The median age was 64 years (Interquartile Range [IQR] 56–70), and 52.1% (365) were female. Our cohort was diverse: 48.2% (338) identified as non-Hispanic (NH) White, 17.4% (122) as Hispanic, 10.8% (76) as NH Black, and 9.4% (66) as Other. For 92.9% (651) of patients, English was the primary language recorded in the EMR. Most patients were publicly insured with Medicare (44.9%) or Medicaid (12.8%), while 41.7% of patients were commercially insured. The median follow-up time for the cohort was 851 days (IQR 759–951).

**Table 1:** Characteristics of Patients Aged 45–75 Years with a Positive Stool-Based Test, 2022–2023

Characteristic	*N* (%)
**Age [median years (Q1**–**Q3)]**	64 (56–70)
**Sex**	
Male	336 (47.9)
Female	365 (52.1)
**Race/ethnicity**	
NH White	338 (48.2)
NH Black	76 (10.8)
Hispanic	122 (17.4)
NH other	66 (9.4)
Unknown	99 (14.1)
**Primary language**	
English	651 (92.9)
Non-English	50 (7.1)
**Insurance**	
Medicare	315 (44.9)
Commercial	292 (41.7)
Medicaid	90 (12.8)
Unknown/self-pay	4 (0.6)
**Body mass index [median (Q1**–**Q3)]**	26.9 (23.4–31.4)
**Charlson Comorbidity Index**	
0	51 (7.3)
1	114 (16.3)
2	169 (24.1)
≥ 3	367 (52.4)
**Prior colorectal cancer screening**	
Any prior CRCS	293 (41.8)
Colonoscopy	219 (31.2)
FIT/FOBT	69 (9.8)
mt-sDNA	56 (8.0)
Sigmoidoscopy	5 (0.7)
CT colonography	1 (0.1)
Number of ambulatory visits* [median visits (Q1–Q3)]	4 (2–10)

^*^Number of ambulatory visits during the year prior to the positive stool test

*CRCS* colorectal cancer screening, *FIT* fecal immunochemical test, *FOBT* fecal occult blood test, *IQR* interquartile range, *mt-sDNA* multi-target stool DNA, *NH* non-Hispanic, *Q1* first quartile, *Q3* third quartile

Our cohort had a substantial comorbidity burden, whereby 52.4% (367) had a CCI score of ≥ 3, 24.1% (169) had a CCI score of 2, 16.3% (114) had a CCI score of 1, and 7.3% (51) had a CCI score of 0. The median BMI in our sample was 26.9 (IQR 23.4–31.4). Prior CRC screening use was observed in 41.8% (293), with colonoscopy as the most common modality (31.2%) followed by FIT/FOBT (9.8%) and multi-target stool DNA (8.0%). Regarding health system utilization, the median number of ambulatory visits in the year preceding the positive stool result was 4 (IQR 2–10 visits).

#### Follow-up After Positive Stool-Based Test

In our cohort, 59.6% (418) of patients completed a colonoscopy within 180 days of a positive stool-based test (Table 2). When evaluating follow-up colonoscopy completion at other time points, 44.5% (312) completed a colonoscopy within 90 days, 68.5% (480) within 365 days, and 74.8% (524) at any time point in our follow-up period. The median time from a positive stool test to follow-up colonoscopy was 109 days (IQR 44–690 days).

**Table 2:** Clinical Characteristics Regarding Follow-up Colonoscopy

Characteristic	*N* (%)
Completed colonoscopy*	
Within 90 days	312 (44.5)
Within 180 days	418 (59.6)
Within 365 days	480 (68.5)
Any time	524 (74.8)
**Time to colonoscopy, entire cohort [median days (Q1–Q3)]**	109 (44–690)
**GI clinic**	
Visit attended	296 (42.2)
**Time to colonoscopy [median days (Q1–Q3)]**	110 (54–329)
Stool result to GI clinic visit	35.5 (15–95)
GI clinic visit to colonoscopy	47 (21–90)
**Direct Access**	
Enrolled	74 (10.6)
**Time to colonoscopy [median days (Q1-Q3)]**	211 (62–735)
Stool result to Direct Access enrollment	10 (4–76)
Direct Access enrollment to colonoscopy	59 (30–134)
Colonoscopy reminder attempts**	
0	329 (63.6)
≥ 1	188 (36.4)
Successful patient outreach***	
No	348 (67.3)
Yes	169 (32.7)
Last outreach to colonoscopy [median days (Q1–Q3)]***	7 (5–20)

^*^Includes CT colonography (*N* = 12) as a modality to evaluate the positive stool-based test

^**^Colonoscopy reminder attempts among patients who completed colonoscopy

^***^Successful patient outreach was defined as evidence that the patient had received a colonoscopy reminder message or telephone call

*GI* gastroenterology, *Q1* first quartile, *Q3* third quartile

**Figure 1:**
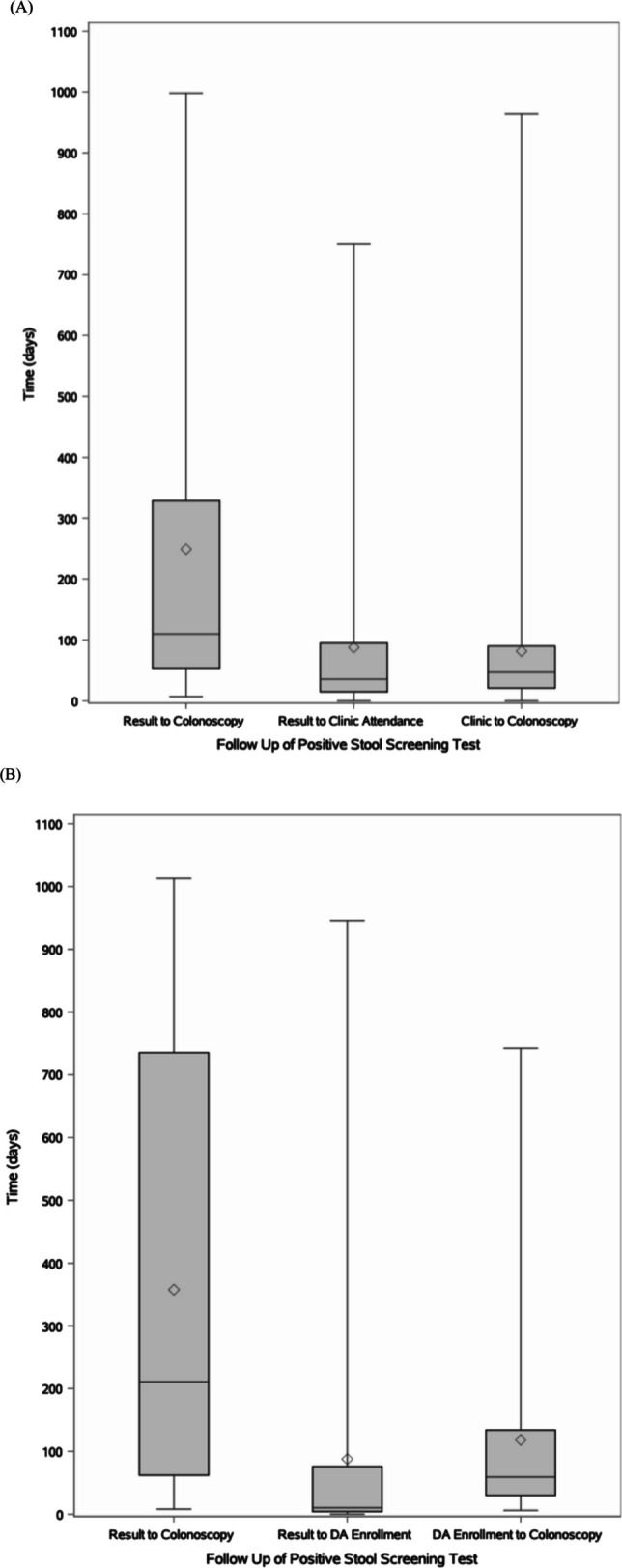
Follow-up of positive stool screening test. (A) GI clinic patients. (B) Direct Access patients.

Among all patients, 42.2% (296) attended the GI clinic following the positive stool test. Among individuals who attended the GI clinic, the median time from the positive stool test to follow-up colonoscopy was 110 days (IQR 54–329) (Fig. 1). The median time from the positive stool test to the date of GI clinic attendance was 35.5 days (IQR 15–95), while the median time from the date of GI clinic attendance to follow-up colonoscopy was 47 days (IQR 21–90). In our cohort, 10.6% (74) of patients were enrolled in Direct Access, where the median time from the positive stool test to follow-up colonoscopy was 211 days (IQR 62–735). The median time from the positive stool test to the date of Direct Access enrollment was 10 days (IQR 4–76), while the median time from the date of Direct Access enrollment to follow-up colonoscopy was 59 days (IQR 30–134).

Among only those patients who completed colonoscopy, 36.4% (188) had at least one colonoscopy reminder attempt and 32.7% (169) were successfully outreached. For those with documented reminder attempts, 110 individuals received one, 40 received two, 26 received three, 10 received four, and 2 received five reminders. There was a median of 7 days (IQR 5–20) between the date of last outreach and the colonoscopy.

### Factors Associated with Timely Colonoscopy and Barriers to Timely Completion

In our multivariable model, successful outreach increased timely colonoscopy completion by 52% (HR 1.52, 95% CI 1.21–1.91), whereas patients enrolled in Direct Access were 40% less likely (HR 0.59, 95% CI 0.41–0.86) to complete timely colonoscopy (Table 3). Sensitivity analyses that examined colonoscopy completion at 90 days, 365 days, and any time point during follow-up showed similar findings (Supplementary Table 1). While GI clinic attendance decreased the likelihood of colonoscopy completion within 90 days (HR 0.72, 95% CI 0.56–0.91), this effect was no longer observed after 90 days. There was no association observed with any sociodemographic factors (i.e., age, sex, race/ethnicity, insurance, comorbidity status).

**Table 3:** Cox Proportional Hazards Models to Evaluate Factors Associated with Timely Colonoscopy Completion (Within 180 Days of a Positive Stool Test)

		**Univariate**	**Multivariable**
**Variable**	***N***	**HR (95% CI)**	**HR (95% CI)**
**Age**			
10 year increment	701	1.00 (0.88–1.11)	1.19 (0.96-1.47)
**Sex**			
Female	365	Reference	Reference
Male	336	0.82 (0.68–0.99)	0.85 (0.70–1.03)
**Race/ethnicity**			
NH White	338	Reference	Reference
NH Black	76	0.89 (0.64–1.23)	1.00 (0.71–1.40)
Hispanic	122	0.99 (0.76–1.28)	1.08 (0.82–1.43)
NH other	66	0.85 (0.60–1.20)	0.82 (0.58–1.17)
Unknown	99	0.90 (0.67–1.21)	0.92 (0.69–1.24)
**Insurance**			
Medicare	315	Reference	Reference
Commercial	292	1.14 (0.93–1.40)	1.24 (0.94–1.64)
Medicaid	90	0.89 (0.65–1.22)	1.04 (0.72–1.50)
Unknown/self-pay	4	1.97 (0.63–6.16)	1.39 (0.43–4.47)
**Charlson Comorbidity Index**			
0	51	Reference	Reference
1	114	0.88 (0.58–1.32)	0.82 (0.53–1.26)
2	169	0.91 (0.62–1.34)	0.79 (0.49–1.28)
≥ 3	367	0.77 (0.54–1.11)	0.66 (0.39–1.11)
**GI clinic visit attended**			
No	405	Reference	Reference
Yes	296	1.04 (0.85–1.26)	0.93 (0.75–1.14)
**Enrolled in Direct Access**			
No	627	Reference	Reference
Yes	74	0.70 (0.49–0.98)	0.59 (0.41–0.86)
**Successful patient outreach**			
No	524	Reference	Reference
Yes	177	1.34 (1.09–1.66)	1.52 (1.21–1.91)

**Figure 2:**
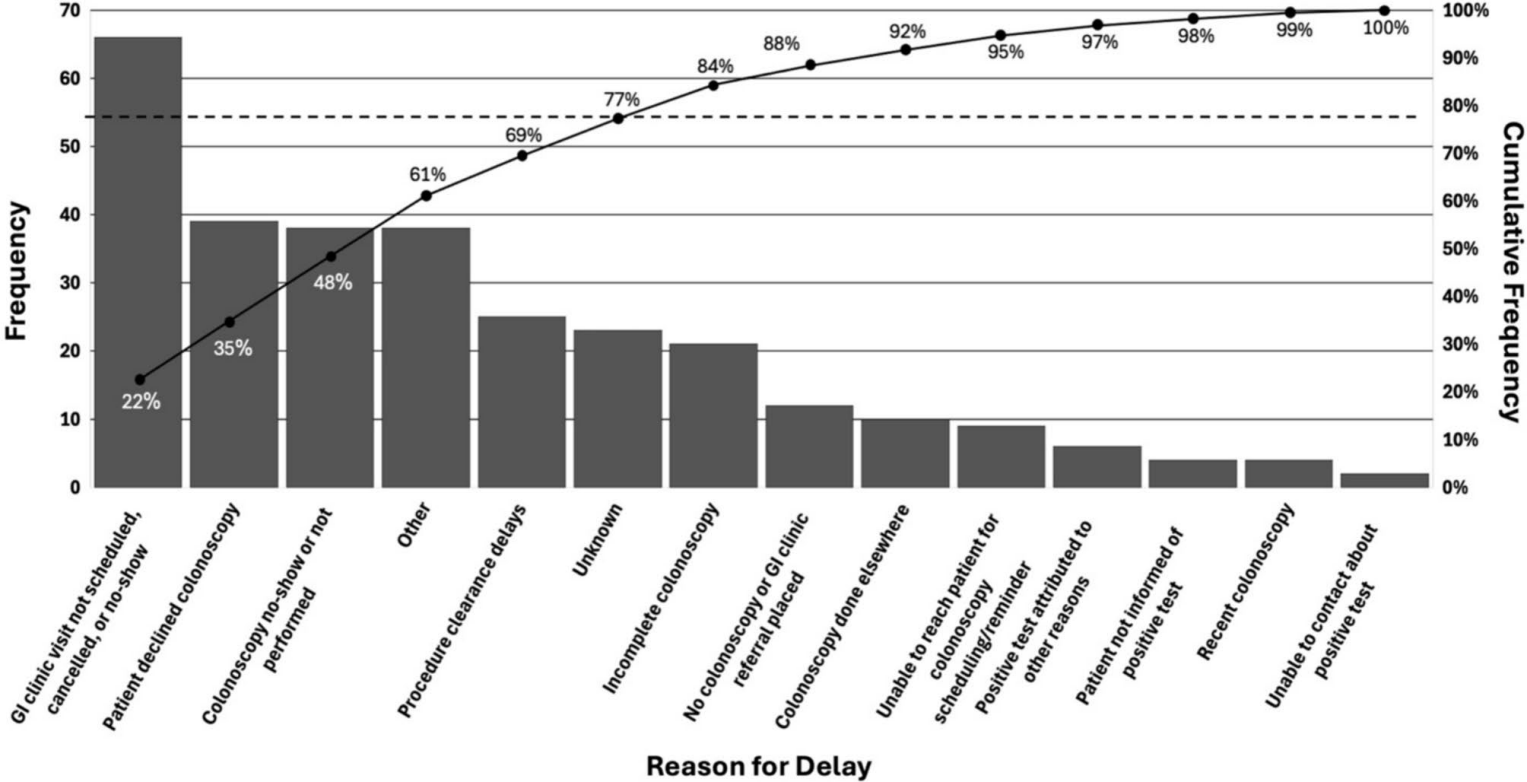
Pareto analysis of barriers to timely colonoscopy completion

Among the 25.2% (177/701) of patients who did not complete a colonoscopy during our follow-up period, 24.3% (43) attended GI clinic and 10.7% (19) were enrolled in Direct Access. Our Pareto analysis revealed that the most common reasons for lack of timely colonoscopy were (1) GI clinic visit not scheduled or not attended (22.1%), (2) patient refusal of colonoscopy (13.1%), and (3) colonoscopy no-show or cancellation (12.8%) (Fig. 2).

## DISCUSSION

In an urban, integrated health system, we found that 59.6% of patients completed a colonoscopy within 180 days of a positive stool-based result, with the largest increase in colonoscopy completion between 90 and 180 days. Overall, the median time to follow-up colonoscopy fell well within intervals in which an increased risk of CRC has not been demonstrated; however, substantial variability in completion was observed, with a sizeable subset of patients experiencing prolonged delays.^4,5^ At all time points, we found that patient outreach—including reminders and counseling—was consistently associated with colonoscopy completion, underscoring the critical role of navigation in closing the screening loop.

The timing of follow-up colonoscopy has a measurable effect on CRC outcomes.^4,5^ Compared with individuals who received follow-up colonoscopy within 30 days of a positive stool test, those who underwent colonoscopy at 7–12 months had 37% higher odds of any CRC and 55% higher odds of advanced-stage CRC, with the odds nearly doubling when colonoscopy was delayed beyond 12 months.^4^ CRC mortality also rises with colonoscopy delays; one study found a 52% higher risk of death among individuals with colonoscopy at 19–21 months versus those who received it within 3 months of an abnormal stool test.^5^ Consequently, there is a growing recognition among national societies of the need for benchmarks around timely colonoscopy, with a HEDIS measure currently under development.^2,21^

Our report falls just short of the 80% target for completion and is consistent with an analysis of administrative claims and EMR data from nearly 40 different health care organizations with follow-up colonoscopy rates of 43.2%, 51.4%, and 56.1%, at 90 days, 180 days, and 365 days, respectively.^7^ Consistent with the literature, most subjects in our study completed follow-up colonoscopy within 6 months, which may represent a critical window for addressing barriers and enhancing colonoscopy uptake. Other studies of integrated health systems demonstrate varying completion at 3 months (51%–73%), 6 months (63%–81%), and 12 months (68%−84).^19^ In contrast to our study population, these samples were predominantly younger (< 65 years old), commercially insured, and healthier (CCI of 0). Our cohort, however, was older (median age 64), more diverse (less than 50% non-Hispanic White), primarily publicly insured, and had multiple comorbidities (over half with a CCI score ≥ 3).

Referring patients directly to colonoscopy without the intervening GI office visit has been considered a means to expedite colonoscopy completion. By contrast, we found that Direct Access enrollment was negatively associated with colonoscopy completion at all timepoints. While GI clinic attendance was associated with lower colonoscopy completion at 90 days (likely due to wait times associated with establishing specialty care), this effect was not significant beyond 3 months, and time to colonoscopy was shorter for patients who attended GI clinic compared to Direct Access enrollees (median of 110 vs. 211 days, respectively). Our Pareto analysis revealed that in over half of the cases where colonoscopy was delayed beyond 180 days, the primary reasons were (1) GI clinic visit was not scheduled/missed, (2) the patient declined colonoscopy, (3) colonoscopy no-show or cancellation, and (4) medical clearances were not obtained. These findings are consistent with previous studies in Veterans Affairs systems, federally qualified health centers, and safety-net settings.^8,10,18,20,22^

A system that accelerates colonoscopy scheduling for high-priority indications is certainly beneficial. Notably within the Direct Access pathway, even as median times to enrollment (10 days) and subsequent colonoscopy (59 days) do not signal excessive delay, a number of patients experienced delays in both segments and the sum of these timepoints reveals the overall impact (median time to colonoscopy of 211 days, IQR 62–735). Our results suggest that among older and sicker patients, additional resources are necessary to facilitate timely follow-up (e.g., a provider who can coordinate required procedural clearances). While most intervention studies examined the impact of navigation or organizational initiatives, patient-centered strategies that educate individuals about the importance of follow-up colonoscopy and address both cognitive and affective barriers are also critical.^11,23–26^ In our cohort, sociodemographic factors did not impact outcomes at any time point, a finding that demonstrates mixed results in the literature.^7,9,19^ Rather, colonoscopy completion is a complex behavior that is shaped by cognitive processing, emotional responses, and sociocultural influences. Examples of such considerations include differences in health literacy regarding the need for colonoscopy, varying levels of self-efficacy with bowel preparation, and fears of the procedure or of potential cancer detection.^11,12^ These decision-making elements are certainly more malleable than fixed demographic factors and should be the target of any educational intervention.

Similar to other studies, we found that system-level practices best accounted for variations in colonoscopy completion.^19,27^ Notably, patients who received at least one reminder were 52% more likely to complete timely colonoscopy, with a median of 7 days (IQR 5–20) between the final reminder and the procedure. The optimal timing and frequency of reminders remain unclear but are important considerations for informing the efforts of patient navigators. Our study did not directly assess the impact of patient navigation; however, a recent randomized trial demonstrated a 13% increase in follow-up colonoscopy completion when navigation was implemented.^25^

While our study has limitations, it also provides valuable real-world insights. We were unable to distinguish between colonoscopies that were not completed and those for which data were missing; consequently, all missing colonoscopy data were categorized as “not completed.” Despite receiving a positive stool test in our health system, some patients may have been referred to or sought specialty care elsewhere. In these instances, the responsibility for tracking colonoscopy completion and for retrieving endoscopy records largely falls on the ordering provider and may be missing from the EMR. Similar to other retrospective studies, sociodemographic information such as sex, race/ethnicity, and language preferences may not be self-reported but determined at the discretion of the entering provider. Although we aimed to analyze each step from a positive stool test to colonoscopy completion, we were unable to capture referrals to GI clinic or Direct Access that occurred outside of orders or provider documentation (i.e., communications through staff messages). In our study, only one-third of patients had recorded reminder attempts or were successfully outreached. However, documentation practices for reminders vary across endoscopy and GI clinic sites. Our study thus emphasizes the critical importance of a centralized framework that (1) integrates standardized workflows, (2) facilitates close coordination across departments, and (3) is tailored to address operational gaps within health systems to ensure abnormal stool tests are followed through colonoscopy completion. Finally, our sample size, which was bounded by the study period, may have limited the ability to demonstrate small differences in risk.

As healthcare organizations evaluate the impact of pandemic-related disruptions and respond to a growing preference for non-invasive screening, it is critical to assess current practices and prioritize efforts to close gaps that delay timely follow-up. In an older population with greater comorbidity burden, we identified system-level factors that impacted timely colonoscopy, while an analysis of colonoscopy delays revealed multiple system- and patient-level elements. Future interventions should seek to augment existing education and navigation initiatives while optimizing institutional infrastructures to improve care coordination and facilitate timely completion.

## Supplementary Information

Below is the link to the electronic supplementary material.

ESM1 (DOCX 22.0 KB)

## Data Availability

The data supporting the findings of this study are available from the corresponding author upon reasonable request.
