# Supplementary material for: Rates and Timing of Follow-up Colonoscopy After a Positive Stool-Based Test in an Integrated Health System
Source: J Gen Intern Med. 2026 Feb 26;41(9):2518–25. doi: 10.1007/s11606-026-10226-8 (PMC13293160; doi:10.1007/s11606-026-10226-8)
Supplement: Supplementary file 1 — (DOCX 22.0 KB) [file 11606_2026_10226_MOESM1_ESM.docx]

**Supplementary Table 1. Sensitivity Analysis to Evaluate Factors Associated with Colonoscopy Completion at (A) 90 days, (B) 365 days, and (C) Any Time Point Following a Positive Stool-Based Test.**

CI = confidence interval, GI = gastroenterology, HR = hazard ratio, NH = non-Hispanic

(A)

| **Characteristic** | **N** | **HR (95% CI)** |
| --- | --- | --- |
| **Age**  10 year increment | 701 | 1.26 (0.99 – 1.62) |
| **Sex**  Female  Male | 365  336 | Reference  0.90 (0.72 – 1.12) |
| **Race/Ethnicity**  NH White  NH Black  Hispanic  NH Other  Unknown | 338  76  122  66  99 | Reference  0.96 (0.65 – 1.42)  1.00 (0.72 – 1.39)  0.72 (0.47 – 1.11)  0.89 (0.63 – 1.25) |
| **Insurance**  Medicare  Commercial  Medicaid  Unknown/Self-Pay | 315  292  90  4 | Reference  1.23 (0.89 – 1.69)  1.11 (0.73 – 1.70)  2.18 (0.67 – 7.08) |
| **Charlson Comorbidity Index**  0  1  2  ≥ 3 | 51  114  169  367 | Reference  0.85 (0.52–1.39)  0.69 (0.39–1.21)  0.56 (0.30–1.03) |
| **GI Clinic Visit Attended**  No  Yes | 405  296 | Reference  0.72 (0.56–0.91) |
| **Enrolled in Direct Access**  No  Yes | 627  74 | Reference  0.53 (0.34–0.83) |
| **Successful Patient Outreach**  No  Yes | 524  177 | Reference  1.56 (1.19–2.03) |

(B)

| **Characteristic** | **N** | **HR (95% CI)** |
| --- | --- | --- |
| **Age**  10 year increment | 701 | 1.14 (0.94 – 1.39) |
| **Sex**  Female  Male | 365  336 | Reference  0.89 (0.74 – 1.06) |
| **Race/Ethnicity**  NH White  NH Black  Hispanic  NH Other  Unknown | 338  76  122  66  99 | Reference  0.98 (0.71 – 1.35)  1.12 (0.87 – 1.45)  0.90 (0.65 – 1.24)  0.93 (0.70 – 1.23) |
| **Insurance**  Medicare  Commercial  Medicaid  Unknown/Self-Pay | 315  292  90  4 | Reference  1.21 (0.93 – 1.57)  1.00 (0.71 – 1.40)  1.49 (0.54 – 4.10) |
| **Charlson Comorbidity Index**  0  1  2  ≥ 3 | 51  114  169  367 | Reference  0.80 (0.53 – 1.19)  0.77 (0.50 – 1.21)  0.70 (0.43 – 1.12) |
| **GI Clinic Visit Attended**  No  Yes | 405  296 | Reference  1.06 (0.88 – 1.29) |
| **Enrolled in Direct Access**  No  Yes | 627  74 | Reference  0.66 (0.47 – 0.92) |
| **Successful Patient Outreach**  No  Yes | 524  177 | Reference  1.72 (1.40 – 2.13) |

(C)

| **Characteristic** | **N** | **HR (95% CI)** |
| --- | --- | --- |
| **Age**  10 years | 701 | 1.12 (0.93 – 1.34) |
| **Sex**  Female  Male | 365  336 | Reference  0.88 (0.74 – 1.04) |
| **Race/Ethnicity**  NH White  NH Black  Hispanic  NH Other  Unknown | 338  76  122  66  99 | Reference  1.06 (0.79 – 1.43)  1.22 (0.96 – 1.56)  0.88 (0.65 – 1.21)  0.87 (0.66 – 1.15) |
| **Insurance**  Medicare  Commercial  Medicaid  Unknown/Self-Pay | 315  292  90  4 | Reference  1.20 (0.93 – 1.55)  0.95 (0.69 – 1.31)  1.31 (0.47 – 3.60) |
| **Charlson Comorbidity Index**  0  1  2  ≥ 3 | 51  114  169  367 | Reference  0.85 (0.58 – 1.26)  0.83 (0.54 – 1.27)  0.74 (0.47 – 1.18) |
| **GI Clinic Visit Attended**  No  Yes | 405  296 | Reference  1.15 (0.96 – 1.38) |
| **Enrolled in Direct Access**  No  Yes | 627  74 | Reference  0.69 (0.50 – 0.93) |
| **Successful Patient Outreach**  No  Yes | 524  177 | Reference  1.88 (1.54 – 2.30) |
